# Is PCR better than culture in TB diagnosis: myth or reality?

**DOI:** 10.1186/1471-2334-14-S3-P46

**Published:** 2014-05-27

**Authors:** Ruqaiyah Johar, Kiran Chawla, Chiranjay Mukhopadhyay

**Affiliations:** 1 Department of Microbiology, Kasturba Medical College, Manipal University, Manipal, India

## Background

Polymerase chain reaction (PCR) is emerging as a new diagnostic tool for tubercular infections. Studies indicate variable results in relation to its efficacy making it as an investigation which is operator dependant. It still remains a dilemma to choose between PCR and culture as the most dependant and reliable diagnostic test. The purpose of our study was to determine the efficacy of PCR in diagnosis of pulmonary and extra-pulmonary tuberculosis and its comparison with conventional culture.

## Methods

In this observational study, 50 pulmonary and 50 extra-pulmonary specimens from suspected tuberculosis patients were included. All samples after initial processing were subjected to microscopy, conventional culture and PCR.

## Results

For pulmonary specimens, positivity of smear, culture and PCR were 56%, 72% and 74%, respectively. Sensitivity and specificity of PCR was found to be 89% and 71% respectively. For extra-pulmonary specimens, positivity of smear, culture and PCR were 36%, 58% and 78%, respectively. Sensitivity and specificity of PCR was found to be 83% and 29% respectively (with culture as gold standard) whereas it was 92% and 67%, respectively (with clinical diagnosis as gold standard). PCR facilitated additional detection of 4% and 20% pulmonary and extra-pulmonary TB cases respectively than by conventional culture. Results of PCR were obtained in 6-8 hours as compared to 6-8 weeks by conventional culture.

## Conclusion

The study highlights PCR as a better diagnostic tool in detection of tuberculosis. Early results of PCR help in early institution of anti tubercular treatment and thus controlling the spread of disease.

